# Alcohol intake and endometrial cancer risk: a meta-analysis of prospective studies

**DOI:** 10.1038/sj.bjc.6605698

**Published:** 2010-05-18

**Authors:** E Friberg, N Orsini, C S Mantzoros, A Wolk

**Affiliations:** 1 Division of Nutritional Epidemiology, The National Institute of Environmental Medicine, Karolinska Institutet, Stockholm, Sweden; 2 Division of Endocrinology, Diabetes, and Metabolism, Department of Medicine, Beth Israel Deaconess Medical Center, Harvard Medical School, Boston, Massachusetts 02215, USA

**Keywords:** alcohol consumption, endometrial cancer risk, meta-analysis

## Abstract

**Background:**

Studies on alcohol intake in relation to endometrial cancer risk have produced inconsistent results.

**Methods:**

For a meta-analysis, we identified cohort studies of alcohol and endometrial cancer by a literature search of Pub-Med and Embase up to 1 March 2010 and by searching the reference lists of relevant articles.

**Results:**

Seven cohort studies, including 1 511 661 participants and 6086 endometrial cancer cases, were included in the dose–response random-effect meta-regression model. Compared with non-drinkers, women drinking less than 1 drink of alcohol (13 g of ethanol) per day had a lower risk for endometrial cancer; this risk was lower by 4% (95% confidence interval (95% CI): 0.93–1.00) for consumption up to 0.5 drink per day and by 7% (95% CI: 0.85–1.02) for consumption up to 1 drink. However, we found evidence of an increased risk for endometrial cancer for intakes higher than two alcoholic drinks per day: compared with non-drinkers, the risk was higher by 14% (95% CI: 0.95–1.36) for 2–2.5 drinks per day and by 25% (95% CI: 0.98–1.58) for >2.5 drinks per day.

**Conclusion:**

Our meta-analysis indicates a possible J-shaped relationship between alcohol intake and endometrial cancer risk.

Alcohol has been claimed to increase the risk of endometrial cancer by increasing oestrogen levels (Gavaler and Van Thiel, 1992; Hankinson *et al*, 1995; Onland-Moret *et al*, 2005; Rinaldi *et al*, 2006), which has been shown to increase endometrial cancer risk (Graham and Clarke, 1997). However, a moderate alcohol intake has also been shown to have a beneficial effect on insulin sensitivity and insulin levels (Davies *et al*, 2002). Hyperinsulinaemia has been shown to stimulate endometrial cell proliferation (Nagamani and Stuart, 1998).

Certain studies have reported an increased risk of endometrial cancer in relation to alcohol intake (La Vecchia *et al*, 1986; Parazzini *et al*, 1995; Terry *et al*, 1999; Setiawan *et al*, 2008); one cohort and one case–control study reported a decreased risk (Webster and Weiss, 1989; Folsom *et al*, 2003), whereas others have not shown a significant association (Williams and Horm, 1977; Cusimano *et al*, 1989; Kato *et al*, 1989; Austin *et al*, 1993; Gapstur *et al*, 1993; Levi *et al*, 1993; Shu *et al*, 1993; Swanson *et al*, 1993; Kalandidi *et al*, 1996; Goodman *et al*, 1997; Newcomb *et al*, 1997; Jain *et al*, 2000; Weiderpass and Baron, 2001; Loerbroks *et al*, 2007; Hosono *et al*, 2008; Kabat *et al*, 2008; Allen *et al*, 2009; Friberg and Wolk, 2009). Of four studies on endometrial cancer or corpus uteri cancer among alcoholics, one showed a decreased risk (Weiderpass *et al*, 2001), whereas the others, which had very few cases, showed no significant results (Adami *et al* 1992; Tonnesen *et al*, 1994; Sigvardsson *et al*, 1996).

To quantify the association between alcohol consumption and endometrial cancer risk, we performed a dose–response meta-analysis of prospective studies.

## Methods

Two persons (E Friberg and N Orsini) independently identified studies by a literature search of the Pub-Med and Embase databases (from their beginning through to 1 March 2010) with the following subject heading terms and/or text words: ‘alcohol’, ‘alcoholic drink’, ‘liquor’, ‘beer’, ‘ethanol’, ‘endometrial cancer’, ‘corpus uteri’. They also reviewed reference lists of the identified publications for additional studies. No language restrictions were imposed.

Seven prospective population-based cohorts have reported on alcohol intake in relation to endometrial cancer risk, and were considered for inclusion in this meta-analysis (Gapstur *et al*, 1993; Terry *et al*, 1999; Jain *et al*, 2000; Folsom *et al*, 2003; Loerbroks *et al*, 2007; Kabat *et al*, 2008; Setiawan *et al*, 2008; Allen *et al*, 2009; Friberg and Wolk, 2009); all reported dose–response data and were included in dose–response analysis. In the case of multiple publications, as from the Iowa Womens Health Cohort (Gapstur *et al*, 1993; Folsom *et al*, 2003), the one supplying dose–response data was chosen (Gapstur *et al*, 1993), and in the case of the National Breast Screening Study in Canada (Jain *et al*, 2000; Kabat *et al*, 2008), the one with the longest follow-up was chosen (Kabat *et al*, 2008).

The data that we extracted included publication data (the first author's last name, year of publication, and country of which the population was studied), number of subjects, follow-up period, risk estimates with their corresponding confidence intervals (CIs), and variables controlled for in the multivariable model. From each study, we extracted the risk estimates that reflected the greatest degree of control for potential confounders.

### Statistical analysis

We examined the relationship between alcohol consumption and endometrial cancer risk on the basis of the relative risks (RRs) and 95% CIs published in each study. We first performed a meta-analysis comparing the highest and the lowest alcohol consumption categories within the specific studies. The summary RR estimate with its 95% CIs were derived with the method of DerSimonian and Laird (1986) by using the assumption of a random-effects model, which incorporated between-studies variability.

We next conducted a dose–response random-effects meta-regression analysis from the correlated natural log of RRs across categories of alcohol intake (Greenland and Longnecker, 1992; Orsini *et al*, 2006). This method requires that the distribution of cases and non-cases or person-time and the RR with its variance estimate for at least three quantitative exposure categories be known. For missing information, we contacted the relevant authors (Jain *et al*, 2000; Kabat *et al*, 2008; Allen *et al*, 2009). As the studies used different units to report alcohol consumption (e.g., grams or number of drinks per day or week), we expressed this as drinks per day, considering 13 g of alcohol to be equivalent to one drink; this corresponds to ∼330 ml of beer, 150 ml of wine, or 45 ml of hard liquor. For each study, the median (Loerbroks *et al*, 2007; Friberg and Wolk, 2009) or mean (Allen *et al*, 2009) level of consumption for each category was assigned to each corresponding RR. When neither was reported, we assigned the midpoint of the upper and lower bound in each category as the average intake (Gapstur *et al*, 1993; Terry *et al*, 1999; Kabat *et al*, 2008; Setiawan *et al*, 2008). If the upper bound in the highest category was not provided, we assumed that it had the same amplitude as the preceding category. We used restricted cubic splines (three knots) to flexibly model and graph the RR (Harrell *et al*, 1988).

In all meta-regression models, statistical heterogeneity between studies was evaluated with Cochran's *Q*-test and the *I*^*2*^ statistic (Higgins and Thompson, 2002), this being the proportion of total variation contributed by between-study variation. Publication bias was assessed by Egger's regression asymmetry test (Egger *et al*, 2001). To investigate potential sources of heterogeneity, we performed a subgroup analysis among studies that adjusted for confounders, such as smoking, use of oral contraceptives, and body mass index. Next, a log-linear estimation of the seven dose–responses was performed. We also conducted a sensitivity analysis iteratively, excluding each study from the overall dose–response meta-analysis.

Statistical analyses were carried out with Stata, version 10 (Stata Corp, College Station, TX, USA). *P*-values that were <0.05 were considered statistically significant. All statistical tests were two sided.

## Results

**Figure 1:**
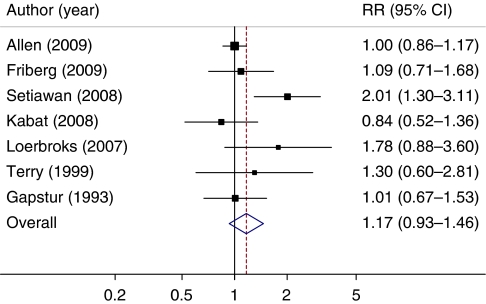
Summary of relative risk (RR) estimates (highest *vs* lowest category) of endometrial cancer risk associated with alcohol consumption. Squares represent study-specific RRs and the sizes of the squares reflect the statistical weight (inverse of the variance) that each study contributed to the summary estimate. Horizontal lines represent 95% confidence intervals (95% CIs), the diamond represents the summary estimate and its 95% CI. Test for heterogeneity *Q*=12.0; *P*=0.061; *I*^*2*^=50%.

Seven independent cohort studies met the predefined inclusion criteria (Table 1), three in North America (Gapstur *et al*, 1993; Kabat *et al*, 2008; Setiawan *et al*, 2008) and four in Europe (Terry *et al*, 1999; Loerbroks *et al*, 2007; Allen *et al*, 2009; Friberg and Wolk, 2009). In total, they included 1 511 661 participants and 6086 endometrial cancer cases, reported dose–response data on alcohol and endometrial cancer risk, and a wide range of alcohol intakes. The comparison of the highest *vs* lowest category of alcohol intake (Figure 1) showed an increase in endometrial cancer cases, although not significantly so (summary RR=1.17; 95% CI: 0.93–1.46); we found no evidence of heterogeneity across studies (*Q*=12.0; *P*=0.061; *I*^*2*^=50%). Furthermore, there was no evidence for publication bias with regard to alcohol and risk. The *P*-value for Egger's regression asymmetry test was 0.26.

Smoking, oral contraceptive use, and body mass index are potentially the most important known confounders of an inverse or of any association between alcohol and endometrial cancer risk. When we restricted the meta-analysis to studies that controlled for these variables (Loerbroks *et al*, 2007; Setiawan *et al*, 2008; Allen *et al*, 2009; Friberg and Wolk, 2009), the summary RR was slightly higher but remained insignificant (summary RR=1.33; 95% CI: 0.92–1.91).

**Figure 2:**
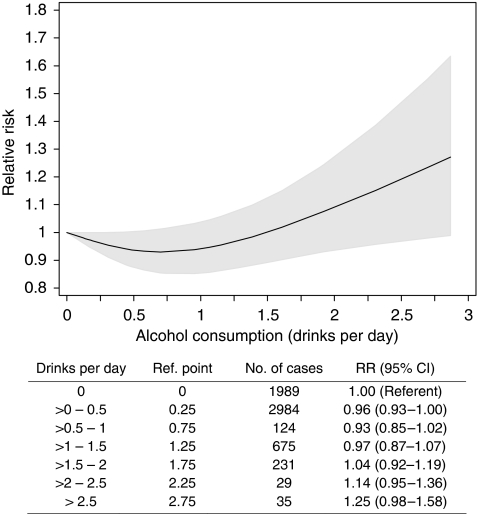
Dose–response relationship between alcohol consumption (drink per day) and endometrial cancer risk estimated with a random-effect meta-regression-restricted cubic spline model. The grey shaded area represents the 95% confidence limits for the fitted curve. Test for heterogeneity *Q*=26.87; *P*-heterogeneity=0.22; *I*^*2*^=6.9%.

In a dose–response analysis, we modelled the relationship between endometrial cancer risk and alcohol consumption using restricted cubic splines (Figure 2) and found some evidence of a non-linear association (*P*-value for linearity=0.01). For exposures less than one drink per day, compared with non-drinkers, risk was lower by 4% (95% CI: 0.93–1.00) for consumption of up to 0.5 drinks per day and by 7% (95% CI: 0.85-1.02) for 0.5 to 1 drink per day (1 drink=13 g of ethanol). Risk seemed to increase after two drinks of alcohol per day, and compared with non-drinkers, it was higher by 14% (95% CI: 0.95–1.36) for 2–2.5 drinks per day and by 25% (95% CI: 0.98–1.58) for 2.5 drinks per day or more (Figure 2). A log-linear estimation of the seven dose–responses rendered a pooled RR of 1.06 (95% CI: 0.94–1.19) (*Q*=13.13; *P*=0.04; *I*^*2*^=54%)

**Figure 3:**
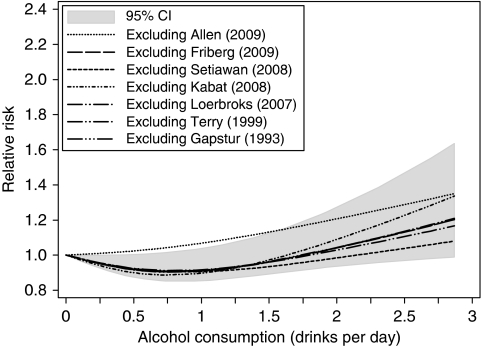
Sensitivity analysis of the dose–response relationship between endometrial cancer risk and alcohol consumption, iteratively removing each study from the overall analysis.

In a sensitivity analysis iteratively removing each study from the overall analysis, we detected a curvilinear association with alcohol in all subgroups (Figure 3).

## Discussion

Our findings suggest that alcohol consumption is weakly associated with endometrial cancer risk. Dose–response analyses showed a non-linear association between risk and number of drinks of alcohol per day: consumption of up to 13 g of alcohol per day (one drink) seemed to be weakly protective, whereas exposure to more than two drinks (>26 g of alcohol) per day may increase risk.

Our analysis must be interpreted in the context of the limited available data. Some degree of non-differential misclassification of alcohol exposure is probable, but this would be expected to attenuate the true relationship. As in all meta-analyses, the possibility of publication bias is of concern, but was not suggested by a formal statistical test. Our meta-analysis has several strengths. First, it was based on prospective studies, which are less susceptible to bias (e.g., recall and selection bias). Second, the dose–response analyses included a wide range of alcohol intake.

A relationship between alcohol and risk of endometrial cancer is biologically plausible. Alcohol increases oestrogen levels (Gavaler and Van Thiel, 1992; Hankinson *et al*, 1995; Onland-Moret *et al*, 2005; Rinaldi *et al*, 2006), which in turn have been shown to increase risk by stimulating the proliferation of endometrial cells (Graham and Clarke, 1997). The EPIC (European Prospective Investigation into Cancer and Nutrition), which is the largest study on alcohol consumption and sex-steroid concentrations, observed a statistically significant increase in blood oestrone levels among women consuming on an average approximately two drinks per day or more compared with non-drinkers (Rinaldi *et al*, 2006). Furthermore, an intake of 30 g of alcohol per day has been shown to improve insulin sensitivity and reduce fasting insulin concentrations (Davies *et al*, 2002), thereby potentially decreasing endometrial cancer risk, although higher intakes do not seem to have these effects (Carlsson *et al*, 2005). Insulin has been shown to stimulate the growth of endometrial stromal cells by binding to insulin receptors in the endometrium (Nagamani and Stuart, 1998). Hyperinsulinaemia may also increase levels of free oestrogen through decreasing concentrations of circulating sex hormone-binding globulin (Nestler *et al*, 1991; Kazer, 1995). Finally, hyperinsulinaemia, through decreasing levels of IGFBP-1, increases circulating free IGF-1, which, by binding and activating IGF-1 receptors in the endometrium, stimulates cell proliferation (Corocleanu, 1993; Irwin *et al*, 1993; Ordener *et al*, 1993; Murphy, 1994; Thiet *et al*, 1994; Weiderpass *et al*, 2003). However, we cannot rule out the possibility that part of the lowered RR observed among women drinking up to one drink per day is because the reference category includes former drinkers and women with health problems.

Our results have important public health implications, given the large number of women consuming alcohol, and the increasing incidence of endometrial cancer in Western societies. The results from this meta-analysis suggest a J-shaped association between alcohol intake and endometrial cancer risk. Moderate alcohol consumption might protect against endometrial cancer, whereas high alcohol consumption may increase risk.

## Figures and Tables

**Table 1:** Characteristics of prospective cohort studies of alcohol consumption and endometrial cancer incidence

**Authors, year (ref no.)**	**Study population, country, follow-up period**	**Case/cohort**	**Range of exposure gram alcohol per day**	**RR (95% CI)^a^**	**Controlled variables**
Allen *et al*, 2009	Million Women Study United Kingdom 1996–2006	4118/1 280 296	0–24.4^b^	0.99 (0.85–1.16)	Age, BMI, smoking, PMH, OC, physical activity, socioeconomic status, region of residence
Friberg and Wolk, 2009	Swedish Mammography Cohort 1987–2007	687/61 226	0–12.4^c^	1.09 (0.71–1.68)	Age, BMI, smoking, PMH, OC, parity, age at menarche, age at menopause, diabetes, education, energy
Kabat *et al*, 2008	National Breast Screening Study Canada 1980–2000	426/89 835	0–35^d^	0.84 (0.52 –1.36)	Age, BMI, PMH, OC, parity, age at menarche, menopausal status, education, calories, calcium, raw vegetables
Setiawan *et al*, 2008	Multiethnic Cohort USA 1993–2002	324/41 574	0–30.0^d^	2.01 (1.30–3.11)	Age, BMI, smoking, PMH, OC, parity, age at menarche, age at menopause, diabetes, hypertension, vigorous exercise, education, race, year, study centre
Loerbroks *et al*, 2007	Netherland Cohort 1986–1997	254/1901	0–37.3^c^	1.78 (0.88–3.6)	Age, BMI, smoking, OC, parity, age at first child, age at menopause, hypertension, physical activity
Terry *et al*, 1999	Twin Cohort Sweden 1961–1992	117/11 659	0–9.5^d^	1.3 (0.6–2.8)	Age, weight, parity, physical activity
Gapstur *et al*, 1993	Iowa Womens Health Cohort USA 1986–1990	160/25170	0–6.0^d^	1.0 (0.7–1.6)	Age, BMI, PMH, parity, age at menopause

Abbreviations: RR=relative risk; CI=confidence interval; BMI=body mass index; PMH=postmenopausal hormone use; OC=oral contraceptive use.

a The measure of RR comparing highest *vs* lowest alcohol category is a rate ratio (hazard ratio) in all studies.

b Mean in the lowest–highest category.

c Median in the lowest–highest category.

d Midpoint in the lowest–highest category.
